# Enhancing healthcare quality and management: AI-driven smart consortium practices at Dapeng Medical Group, Shenzhen

**DOI:** 10.3389/fmed.2026.1680509

**Published:** 2026-06-05

**Authors:** Xiaoxiao Quan, Junjie Pan

**Affiliations:** 1 Shenzhen Second People’s Hospital (The First Affiliated Hospital of Shenzhen University), Shenzhen, China; 2 Wuhan University, Wuhan, China

**Keywords:** artificial intelligence, consortium management, informatization construction, medical service quality, smart healthcare consortium

## Abstract

**Objective:**

To evaluate the impact of smart healthcare consortium management practices, utilizing artificial intelligence (AI) to enhance healthcare service quality and operational efficiency at Dapeng Medical Group, Shenzhen.

**Design:**

A case study analysis of smart healthcare initiatives focusing on AI-driven improvements in patient care and management processes.

**Site:**

Dapeng Medical Group, a comprehensive medical institution in Shenzhen, China.

**Participants:**

Patients receiving care within the Dapeng Medical Group, along with healthcare providers and administrative staff involved in the implementation of the smart healthcare consortium.

**Methods:**

The initiative incorporated advanced technologies, including AI, cloud computing, and the Internet of Things (IoT), to streamline healthcare operations. Key performance metrics such as patient satisfaction, waiting times, diagnostic accuracy, and resource utilization were monitored to assess the effectiveness of the smart healthcare consortium. To clarify the effectiveness of Dapeng Medical Group’s artificial intelligence technologies in smart healthcare consortium management, this study compares relevant management data from before AI adoption (2022) and after AI enablement (2025).

**Results:**

The implementation led to a 25% increase in patient satisfaction, 14% reduction in average waiting times and 34% reduction in the consultation duration, a 29% reduction in error rate of online pre-diagnostic precision.

**Conclusion:**

The integration of AI and smart technologies into healthcare management significantly improved service quality, operational efficiency, and patient outcomes at Dapeng Medical Group. These findings suggest that smart healthcare consortia can serve as a model for future healthcare innovations, ensuring high-quality, efficient, and patient-centered care.

## 1 Introduction

The rise of information technology and artificial intelligence has provided strong support for the development of smart healthcare consortia (1–3). The Dapeng Medical Group in Shenzhen fully recognizes this trend and actively promotes informatization and the establishment of smart healthcare consortia. Since the project’s launch, the group has established a solid foundation in electronic medical record adoption and data processing, laying favorable groundwork for subsequent smart healthcare applications.

One of the primary goals of the smart healthcare consortium initiative is to improve the patient’s medical experience. For instance, the implementation of intelligent appointment scheduling systems has reduced average patient waiting times by 14 min, thereby significantly enhancing patient satisfaction. The initiative also emphasizes the integration and sharing of medical resources through a unified medical information management system, which allows for the seamless exchange of medical data and enables doctors to develop more accurate treatment plans based on comprehensive patient information.

In the realm of intelligent diagnostic assistance, the Dapeng Medical Group has introduced an AI-assisted diagnostic system that leverages deep learning algorithms to analyze imaging and pathological data. The introduction of this system has led to a 29% reduction in error rate of online pre-diagnostic precision and 34% reduction in the consultation duration. This advancement not only enhances diagnostic accuracy but also reduces the burden on doctors, shortens diagnosis times, and further elevates the quality of medical services (4, 5).

Moreover, the initiative places significant emphasis on the advancement of intelligent healthcare services. Through systems for intelligent appointment scheduling and queue management, patients can book appointments in advance and avoid long wait times, while hospitals can better manage doctors’ schedules, maximizing the utilization of medical resources. As a result, the efficiency of doctor utilization has improved by 18%, and patient throughput has increased by 23%. Additionally, the introduction of a patient health management platform has enabled patients to record their health data using mobile devices or smart wearables, sharing this data with doctors in real-time (5–8). The use of this platform has led to a significant improvement in chronic disease management efficiency.

In the practice of the healthcare consortium, artificial intelligence technology plays a significant role (9). With its precision diagnosis and treatment characteristics, AI assists doctors in accurate disease diagnosis and treatment planning through learning and analyzing extensive medical data, thereby improving treatment outcomes (10–12). Additionally, AI optimizes resource allocation, analyzing data to allocate medical resources rationally, improving the efficiency and quality of medical services (13–15). Overall, the Dapeng Medical Group’s initiative on smart healthcare consortium management, guided by information technology and AI, serves as a model for enhancing medical service quality and management and provides positive guidance for the future development of smart healthcare.

## 2 Initiative overview

### 2.1 Construction background

As a comprehensive medical institution integrating healthcare, education, and research, Shenzhen Dapeng Medical Group faced a series of systemic challenges. In terms of resource allocation, medical resources were fragmented within the group, with high-quality resources struggling to be effectively diffused to primary-level units. Service coordination was inadequate, characterized by suboptimal operation of the tiered healthcare system and an underdeveloped two-way referral mechanism, which contributed to relatively weak service capacity at the primary level and suboptimal public trust. Management models exhibited significant barriers, with high coordination costs across different levels and departments, and a performance evaluation system that was insufficiently aligned with the goals of the vertically integrated healthcare consortium. More fundamentally, disparities in digitalization levels among member institutions led to information silos and low data interoperability, which not only constrained management efficiency but also posed inherent obstacles to subsequent efforts in advancing intelligent management. The convergence of these issues necessitated innovative breakthroughs, establishing a practical imperative for the subsequent introduction of artificial intelligence technologies.

### 2.2 Construction objectives

The Dapeng Medical Group’s initiative on smart healthcare consortium management focuses on developing a comprehensive informatization strategy for hospitals, adhering to both international and domestic standards. The plan incorporates advanced technologies, including cloud computing, big data, the Internet of Things, mobile internet, blockchain, and artificial intelligence. The goal is to substantially improve resource allocation efficiency while reducing operational costs. By enhancing scientific management practices and the quality of medical services, the initiative aims to deliver more efficient and high-quality care. Furthermore, it seeks to align with global and local healthcare markets, ensuring sustainable development and fostering the creation of a patient-centered, intelligent healthcare consortium centered on electronic medical records.

## 3 Implementation and achievements of the initiative

To clarify the effectiveness of Dapeng Medical Group’s artificial intelligence technologies in smart healthcare consortium management, this study compares relevant management data from before AI adoption (2022) and after AI enablement (2025).

### 3.1 Application of artificial intelligence technology in smart healthcare consortium management

The application of artificial intelligence (AI) technology within the smart healthcare consortium has brought significant achievements to the Dapeng Medical Group, with notable improvements in patient experience, operational efficiency, and data processing capabilities. Since the implementation of the smart healthcare consortium initiative, patient satisfaction has significantly increased from 68.75% to 93.75%, reflecting a 25-percentage-point growth (Table 1). This improvement highlights the optimization of healthcare service quality and greater patient recognition of their medical experiences. In terms of operational efficiency, the average waiting time decreased from 22.74 min to 19.62 min, a reduction of 14%, while the average consultation duration dropped from 67.57 min to 44.63 min, a 34% reduction. These figures demonstrate smoother patient flow throughout the entire care process and more efficient allocation of medical resources.

**TABLE 1:** Improvement metrics from the implementation of the smart healthcare consortium.

Metric	Before implementation (2022)	After implementation (2025)	Improvement (%)
Patient satisfaction of smart healthcare consortium	68.75%	93.75%	+25%
Average waiting time	22.74 min	19.62 min	−14%
Average consultation duration	67.57 min	44.63 min	−34%
Utilization of electronic medical records	71%	100%	+ 30%
Data processing speed	≤2.98 min	≤1.66 min	+ 44%
Error rate of online pre-diagnostic precision	0.17%	0.12%	−29%
Chronic disease management outcomes	65%	78%	+ 38%
Payment receipt confirmation cycle	35 days	15 days	−20 days

Another highlight of AI technology in the smart healthcare consortium is the improvement in data processing efficiency. AI-driven analysis reduced end-to-end data analysis time from 15.54 min to 8.2 min, a 47% increase in efficiency (Table 2). Additionally, the time required for routine data processing decreased from no more than 2.98 min to no more than 1.66 min, improving by 44% (Table 1). These advancements have significantly shortened the time from data generation to decision support, enabling managers to respond more quickly and improving the overall management efficiency of the consortium.

**TABLE 2:** AI-driven performance enhancements in the smart healthcare consortium.

Application area	Before implementation (2022)	After implementation (2025)	Performance enhancement (%)	AI contribution
Data analysis speed	15.54 min	8.2 min	+47%	Accelerated by AI
Average time to reach diagnosis	90.31 min	64.25 min	−29%	Reduced due to AI application
Efficiency of doctor utilization	72.13%	96.56%	+ 24%	Improved through AI-driven scheduling
Patient throughput	1.09M	1.51M	+ 38.5%	Increased due to AI and smart scheduling
Patient health management platform adoption	12.4%	77.4%	65% participation	Active usage increased

AI has also played an important role in clinical decision support. For instance, the average time to reach a diagnosis decreased from 90.31 min to 64.25 min, a reduction of 29% (Table 2). The extensive application of AI-assisted triage, intelligent medical record reviews, and decision-support algorithms not only accelerated diagnostic processes but also reduced the risk of misdiagnosis and missed diagnoses, further enhancing medical quality and patient safety.

### 3.2 Impact of smart healthcare consortium construction on consortium management

The construction of the smart healthcare consortium has profoundly influenced the management model and resource coordination capabilities of the consortium, particularly in terms of medical resource utilization and patient participation. Through an AI-optimized intelligent scheduling system, doctor utilization increased from 72.13% to 96.56%, a 24% improvement (Table 2). This change ensured the rational allocation of medical resources across different time periods and institutions, reducing idle time and waste. Simultaneously, patient throughput increased significantly, from 1.09 million to 1.51 million, representing a 38.5% growth (Table 2). These figures demonstrate that the construction of the smart healthcare consortium has maximized the efficiency of resource utilization.

Additionally, the construction of the smart healthcare consortium has greatly enhanced patient participation in healthcare services. The adoption rate of the patient health management platform increased from 12.4% to 77.4%, a growth of 65 percentage points (Table 2). This growth signifies that more patients have joined a health management system centered on digital platforms, enabling chronic disease monitoring and personalized medical interventions. This digital healthcare participation model not only benefits patients but also provides more comprehensive data support for medical decision-making.

In terms of financial and operational management, the construction of the smart healthcare consortium has also brought positive changes. The payment receipt confirmation cycle was reduced from 35 days to 15 days, compressing the timeframe by 20 days (Table 1). This improvement optimized the consortium’s cash flow, reduced financial pressures, and improved the efficiency of revenue operations. These changes provide a solid foundation for the sustainable development of the entire consortium.

### 3.3 Practice and achievements of the Dapeng Medical Group’s initiative on smart healthcare consortium management

The implementation of the Dapeng Medical Group’s smart healthcare consortium management initiative has achieved remarkable results in improving healthcare service quality and patient health outcomes, particularly in chronic disease management and diagnostic accuracy. Chronic disease management outcomes increased from 65% to 78%, a growth of 38% (Table 1). This improvement was made possible by the widespread use of the health management platform and AI’s real-time analysis and intervention support for patient health data, enabling more effective monitoring and improvement of chronic disease patients’ health conditions.

In terms of healthcare service quality, the application of AI significantly reduced the error rate in online pre-diagnosis from −0.17% to −0.12%, a 29% relative improvement (Table 1). This result demonstrates the critical role of AI in enhancing early diagnostic accuracy and reducing the risk of misdiagnosis, while also providing higher precision support for subsequent medical procedures.

Moreover, the smart healthcare consortium management initiative has optimized information sharing and collaboration capabilities, enhancing cross-institutional cooperation. With the interconnection of information platforms, medical institutions within the consortium can efficiently share patient data, simplifying referral processes and improving follow-up plans. This cross-institutional collaboration ability is particularly valuable for complex cases and chronic disease management that require multidisciplinary cooperation, providing patients with a more complete and continuous healthcare service network. In addition, the Dapeng Medical Group’s smart healthcare consortium management initiative has delivered outstanding results across multiple dimensions, including improved patient satisfaction (93.75%), optimized resource utilization (doctor utilization at 96.56%, patient throughput at 1.51 million), and significant advancements in chronic disease management and diagnostic accuracy.

Overall, the implementation of the Dapeng Medical Group’s initiative on smart healthcare consortium management, driven by information technology and AI applications, has achieved significant results. Through measures such as optimizing management processes, improving work efficiency, and reducing costs, the overall management level of the healthcare consortium has been elevated. By providing more convenient and efficient medical services and enhancing synergy with other medical institutions, patients receive higher-quality medical experiences and continuous medical services, laying a solid foundation for the future development of the smart healthcare consortium.

## 4 Conclusion and outlook

The Dapeng Medical Group’s initiative on smart healthcare consortium management has provided essential technical support for consortium management and the improvement of medical service quality. Looking forward, the group plans to integrate blockchain technology to further secure patient data and reduce data breaches by 28%. Through AI technology and informatization applications, the consortium has achieved significant improvements in medical service systems, clinical informatization, and regional business synergy. However, smart healthcare consortium construction still faces challenges in technology security, privacy, and other areas, which require ongoing research and exploration.

In the future, smart healthcare consortium construction will continue to evolve to meet higher medical service needs and management requirements. The Dapeng Medical Group will continue to explore and practice using AI technology as a core to promote medical consortium informatization, enhance medical service quality and management levels, and provide better medical services to patients.

### 4.1 Continuously optimize smart healthcare consortium construction

The Dapeng Medical Group will continuously optimize smart healthcare consortium construction, further enhancing medical service quality and management efficiency. The consortium will continue to promote informatization construction, improve the electronic medical records system, elevate clinical informatization application levels, optimize medical service processes, and enhance the patient’s medical experience.

### 4.2 Strengthen the application of artificial intelligence technology

The Dapeng Medical Group will further strengthen the application of artificial intelligence technology to enhance medical service quality and management efficiency. The consortium will utilize AI technology for intelligent data analysis, clinical decision support, medical record quality control, etc., to improve medical quality and patient safety.

### 4.3 Enhance collaboration with regional and external medical institutions

The Dapeng Medical Group will further strengthen collaboration with regional and external medical institutions, achieving effective information sharing and collaboration through the interconnection of information platforms and internet technology, providing cross-institutional continuous medical services.

### 4.4 Face challenges and actively explore solutions

Smart healthcare consortium construction still faces challenges in technology, security, privacy, etc. The Dapeng Medical Group will actively explore solutions to ensure the safe, stable, and efficient operation of the smart healthcare consortium.

Overall, the implementation of the Dapeng Medical Group’s initiative on smart healthcare consortium management has provided essential technical support for consortium management and the improvement of medical service quality. In the future, the Dapeng Medical Group will continue to explore and practice, promoting the intelligent development of the medical consortium and providing better medical services to patients.

## Data Availability

The original contributions presented in this study are included in the article/supplementary material, further inquiries can be directed to the corresponding author.
